# Integrated transcriptome and proteome revealed that the declined expression of cell cycle-related genes associated with follicular atresia in geese

**DOI:** 10.1186/s12864-022-09088-1

**Published:** 2023-01-16

**Authors:** Wanli Yang, Xingyong Chen, Zhengquan Liu, Yutong Zhao, Yufei Chen, Zhaoyu Geng

**Affiliations:** 1 College of Animal Science and Technology, Anhui Agricultural University, No. 130 Changjiang West Road, Hefei, 230036 China; 2 Anhui Province Key Laboratory of Local Livestock and Poultry Genetic Resource Conservation and Bio-breeding, Anhui Agricultural University, NO. 130 Changjiang West Rd, Hefei, 230036 China

**Keywords:** Goose, Atretic follicle, Cell cycle, Matrix metalloproteinase

## Abstract

**Background:**

Geese exhibit relatively low reproductive performance, and follicular atresia is an important factor that restricts the egg production of geese. Systematic analysis of the regulation of follicle atresia in geese through transcriptome and proteome levels could provide meaningful information on clarifying the mechanism of follicle atresia in poultry.

**Result:**

The granulosa cell layer was loose, disintegrated and showed apoptosis in atretic follicles and remained intact in normal follicles. The hormone levels of FSH and LH were significantly decreased in the atresia follicles compared to the normal follicles (*P* < 0.05). A total of 954 differentially expressed genes (DEGs, 315 increased and 639 decreased) and 161 differentially expressed proteins (DEPs, 61 increased and 100 decreased) were obtained in atresia follicles compared to normal follicles, of which, 15 genes were differentially expressed in both transcriptome and proteome. The DEGs were mainly enriched in sodium transmembrane transport, plasma membrane, and transmembrane transporter activity based on the GO enrichment analysis and in the cell cycle pathway based on the KEGG enrichment analysis. The DEPs were mainly enriched in localization, lysosome, and phospholipid-binding based on the GO enrichment analysis. Candidate genes Smad2/3, Smad4, Annexin A1 (*ANXA1*), Stromelysin-1 (*MMP3*), Serine/threonine-protein kinase (*CHK1*), DNA replication licensing factor (*MCM3*), Cyclin-A2 (*CCNA2*), mitotic spindle assembly checkpoint protein (*MAD2*), Cyclin-dependent kinase 1 (*CDK1*), fibroblast growth factor 12 (*FGF12*), and G1/S-specific cyclin-D1 (*CCND1*) were possibly responsible for the regulation of atresia.

**Conclusion:**

The cell cycle is an important pathway for the regulation of follicular atresia. Sodium outflow and high expression of *MMP3* and *MMP9* could be responsible for structural destruction and apoptosis of follicular cells.

**Supplementary Information:**

The online version contains supplementary material available at 10.1186/s12864-022-09088-1.

## Introduction

Follicular atresia is an important factor restricting egg production performance. In birds, less than 5% of the follicles could complete their development process for mature and ovulation, while the remaining 95% become atretic follicles and eventually get degraded [1]. Goose, especially indigenous breeds, exhibits low egg production performance, which causes a relatively higher cost of goslings and lower marketing numbers as compared to broilers and meat ducks. The prevention of follicular atresia and promotion of the normal development of follicle could help increase egg production. Therefore, understanding the mechanism of follicular atresia in geese and screening key signal pathways or genes that regulate follicular atresia would be helpful for identifying molecular markers for selection of goose with high level of atresia.

Follicular atresia is a spontaneous process in which follicles naturally degenerate without an inflammatory response and is generally considered to be caused by defects in the oocyte itself or a lack of necessary growth factors and hormones [2]. The *TGFβ* superfamily plays an important role in follicular development, in which, *BMP-4* and *BMP-7* are positive regulators in the transition from the primordial to the primary follicle, while the anti-Mullerian hormone (*AMH*) is a negative regulator for this transition [3]. The *bFGF* inhibits follicular atresia through the *PI3K-Akt* pathway in chickens [4], while *FGF10* promotes follicular atresia by decreasing the secretion of estradiol in cattle [5]. In bovine, *FGF18* inhibited the secretion of estradiol and progesterone, alters the progression of the cell cycle, and promotes follicular atresia by accelerating the apoptosis of granulosa cells [6]. Hormones have been reported to be involved in follicular development, ovulation, atresia, and other processes. The follicle-stimulating hormone (FSH) could prevent follicular atresia through inhibiting the apoptosis of granulosa cells by inducing their differentiation, the formation of luteinizing hormone receptors, and the synthesis of follicular steroid hormones [7]. It has been proven that atretic follicles exhibited a reduced production of estradiol in granulose cells of porcine [8].

Atretic follicle showed shrunken surface with hemorrhagic spots. The histological and ultrastructural analysis of atretic follicles in two fish breeds revealed the degradation of mitochondria and marked phagocytic activity of digestive vacuoles, myelin, and lipofuscin granules [9]. Granulosa cells were considered to be the first ones to undergo apoptosis in the follicle, which then induced the overall apoptosis and atresia [10]. The atresia of monkey follicles was characterized by three consecutive stages including morphological alterations, cessation of the proliferation and apoptosis, and fragmentation of DNA in granulosa cells that occurred only in late atresia [11]. Furthermore, the follicular development or atresia is thought to be regulated by the survival and apoptosis of granulosa cells in mammals [12]. Unlike the granulosa cells of mammals, which are scattered in the follicular fluid, follicles of poultry have distinct layers of granulosa cells, which make them useful experimental models for the study of follicular atresia.

Many reports have stated the mechanism about follicular atresia in mammals, fish, however, still less was conducted on the systematic research of the molecular mechanism of follicular atresia in poultry. In this study, the histology and reproductive hormones level were compared in normal and atretic follicles. The site of apoptosis of the atretic follicle was first determined in goose. Granulose cells were used for integrated transcriptome and proteome analyses to interpret the molecular mechanism of follicular atresia in geese.

## Results

### Histology and hormone levels of atretic and normal follicles

**Fig. 1:**
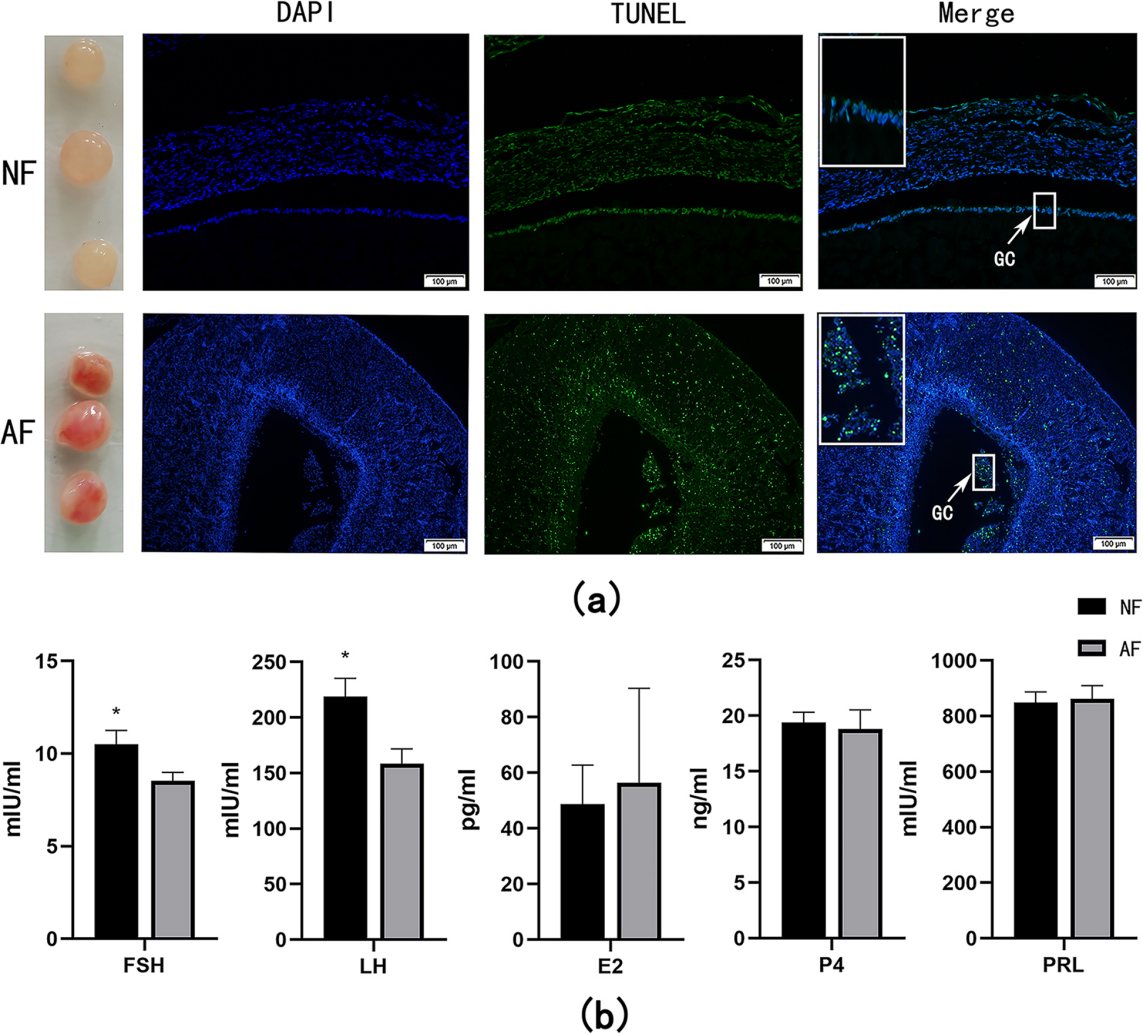
**a** The TUNEL section of normal and atretic follicles. GC, granulosa cells. Follicles were large white follicles (**b**). The expression of reproductive hormones in normal and atretic follicles. FSH, follicle-stimulating hormones; LH, luteinizing hormone; PRL, prolactin; E2, estradiol; P4, progesterone; * = *p* < 0.05, The levels of hormones were compared between normal and atretic follicles

The granulosa cell layer was loose and disintegrated in atretic follicles and remained intact in normal follicles (Fig. 1a). Apoptosis was observed in all parts of atretic follicles and was mostly exhibited in the collapsed granulosa cell layer, which was not observed in the normal follicles (Fig. 1a). The hormone levels of FSH and LH were significantly decreased in the atresia follicles compared to the normal follicles (*P* < 0.05). The levels of hormones E2, P4, and PRL showed no significant differences between atretic and healthy follicles (Fig. 1b).

### De novo transcriptome analysis

**Fig. 2:**
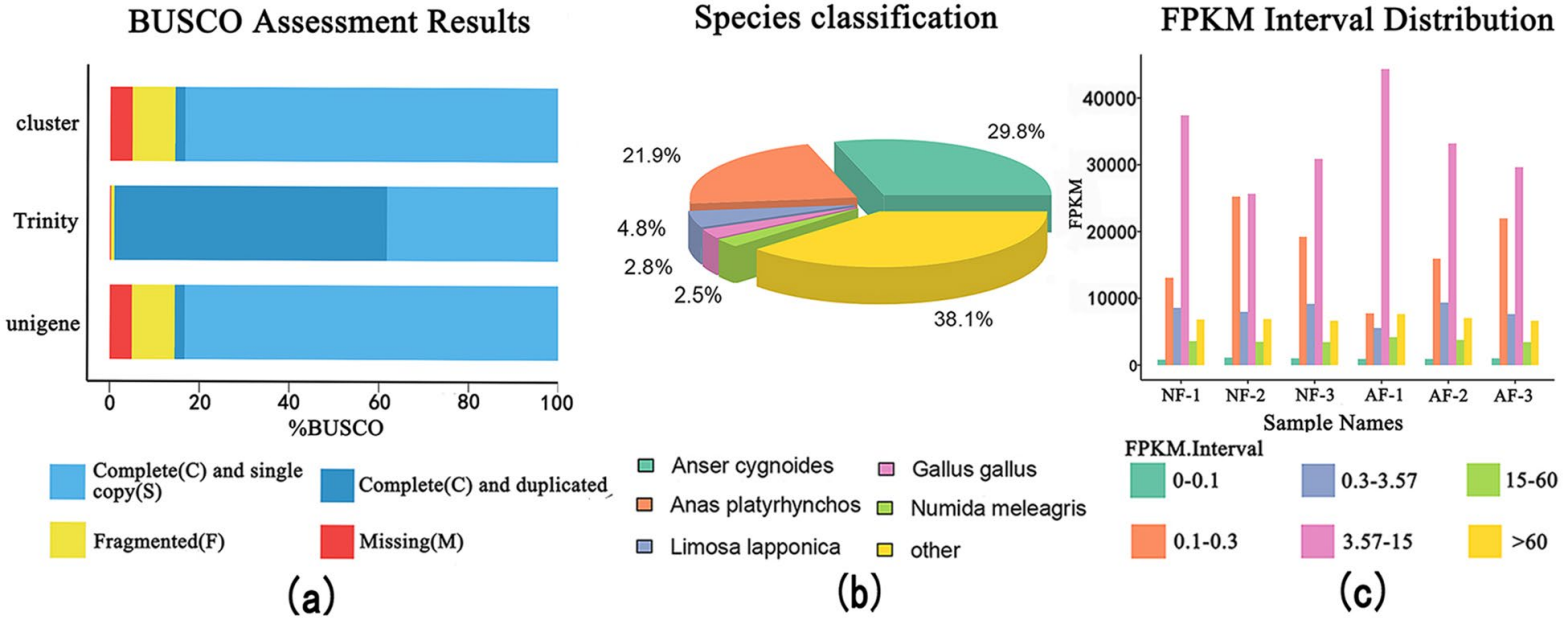
**a** The results of the Benchmarking Universal Single-Copy Orthologs (BUSCO) assessment. **b** Species classification of BLAST analysis of the Nr database. **c** The distribution of FPKM

A total of 141,051,229 raw reads and 135,590,434 clean reads were obtained. The error rates of sequencing were < 0.03, the Q20 and Q30 were > 90%, and the mapped rate was > 80% (Supplementary Table S1). After de novo assembly, 140,475 transcripts and 70,318 unigenes with a mean length of 2007 bp and 1436 bp, respectively, were obtained (Supplementary Table S2). The complete single-copied genes accounted for 83.3% of the all Benchmarking Universal Single-Copy Orthologs (BUSCO), indicating a qualified transcriptome assembled (Fig. 2b). A total of 46,696 unigenes were annotated, and among the seven databases, the Nr database had the highest annotations as compared to other six databases (Supplementary Table S3). According to the results of gene annotation, *Anser cygnoides* showed the highest similarity with the geese among the annotated species (Fig. 2a). The expression levels of all genes were expressed in FPKM values (Supplementary Table S4) and were mainly distributed in the range of 3.57–15 (Fig. 2c).

**Fig. 3:**
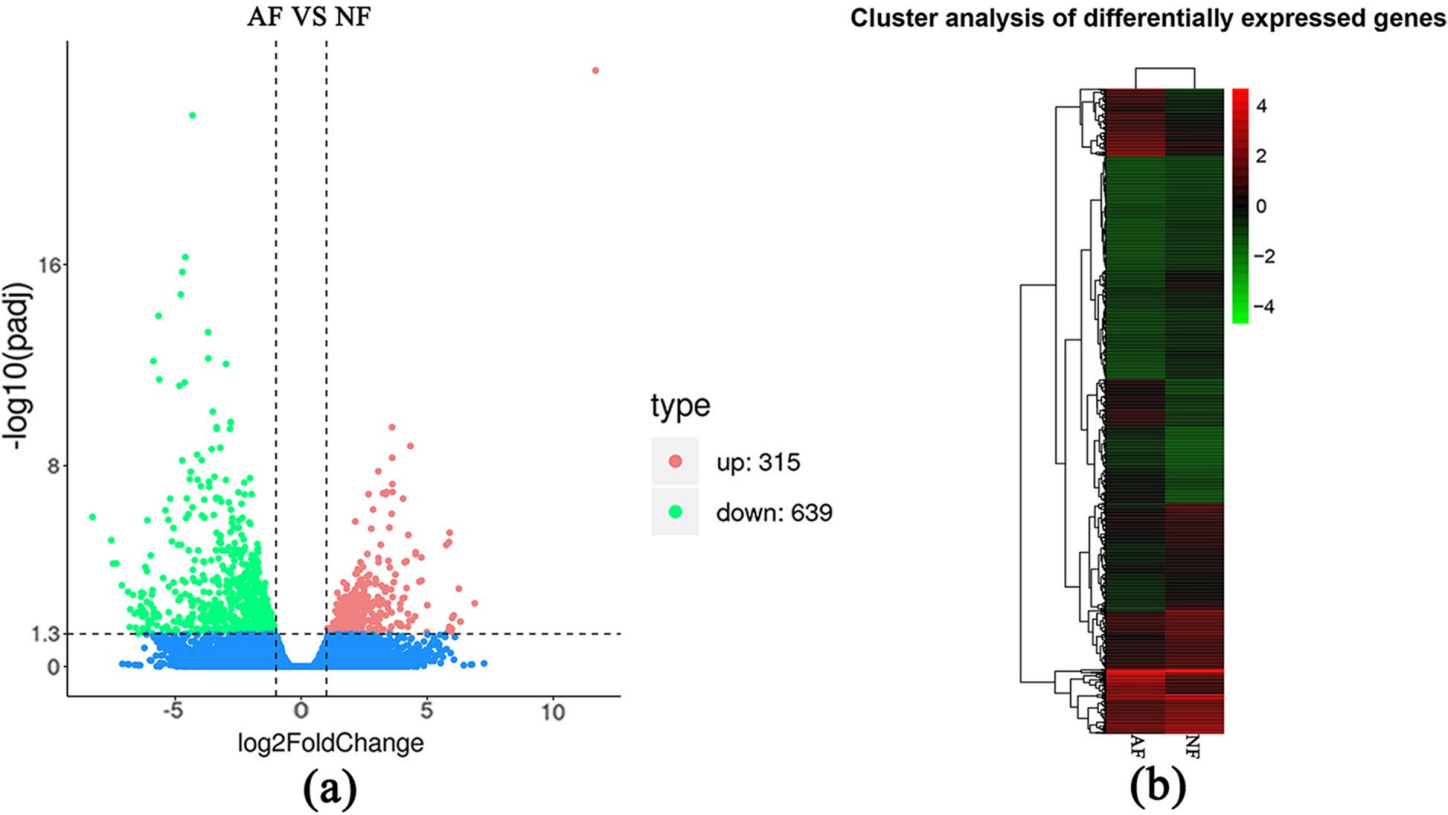
**a** Volcano plot of differentially expressed genes (DEGs) in AF vs NF. AF: atretic follicles; NF: normal follicles. **b** Heatmap of the hierarchical clustering of DEGs in atretic and normal follicles

The principal component analysis (PCA) indicated a significant difference in mRNA expression between AF and NF groups (Fig. S1). A total of 954 DEGs were obtained, of which, 315 were upregulated, and 639 were downregulated in AF when compared to NF (Fig. 3a & b). A detailed analysis of the top 10 DEGs showed that matrix metallopeptidase-3 (*MMP3*), which was responsible for proteolysis, was 11.68 times upregulated in AF (Table 1), and matrix metallopeptidase-7 (MMP7), a disintegrin, metalloproteinase with thrombospondin motifs 18 (*ADAMTS18*) were the matrix metalloproteinases all present in the top 10 upregulated genes (Supplementary Table S5). The downregulated gene with the highest change was the Kazal-type serine protease inhibitor domain-containing protein 1-like gene (log2FC = − 6.19), which is responsible for the regulation of cell growth (Table 1). In addition, growth factors like fibroblast (*FGF12*) and platelet-derived growth factor subunit A (*PDGFA*) were also downregulated in AF (Supplementary Table S5).

**Table 1:** The top 10 upregulated or downregulated mRNAs of goose AF vs NF follicles

Nt ID^**a**^	Nt Description^**b**^	Symbol	UP/DOWN	log2FC^**c**^
XM_005010500	collagenase 3-like	*MMP3*	UP	11.68
XM_027450263	epiplakin	*–*	UP	6.05
XM_013191692	polyketide synthase-nonribosomal peptide synthetase-like	*–*	UP	5.98
XM_027460548	prosaposin-like isoform X1	*PSAP*	UP	5.89
XM_013178059	matrilysin	*MMP7*	UP	5.87
XM_013176668	A disintegrin and metalloproteinase with thrombospondin motifs 18 isoform X2	*ADAMTS18*	UP	5.85
XM_021275530	cysteine and glycine-rich protein 3 isoform X1	*CSRP*	UP	5.75
XM_013191692	highly reducing polyketide synthase PKS6-like	*–*	UP	4.77
XM_027459032	chloride anion exchanger	*–*	UP	4.77
XM_013183939	protein S100-G	*S100G*	UP	4.69
XM_013179956	solute carrier family 2, facilitated glucose transporter member 9	*GLUT9*	DOWN	−4.72
XM_013184728	uncharacterized protein LOC104832909	*–*	DOWN	−4.78
AC189035	transcription factor dp-1-hypothetical protein	*–*	DOWN	−4.83
XM_013194975	endothelin-converting enzyme-like 1	*ECEL1*	DOWN	−4.85
XM_021379024	sodium/iodide cotransporter, partial	*SLC5A5*	DOWN	−4.89
XM_013181091	glycerol kinase	*–*	DOWN	−5.39
XM_027678824	solute carrier family 23 member 3	*IHH*	DOWN	−5.63
XM_013187778	PREDICTED: hemogen	*–*	DOWN	−5.66
XR_001207760	ubiquitin carboxyl-terminal hydrolase 4	*–*	DOWN	−5.92
XM_013178393	kazal-type serine protease inhibitor domain-containing protein 1-like	*–*	DOWN	−6.19

^a^The ID number of a gene, as annotated in the NT database

^b^Description of genes in the NT database

^c^log2FC = log2 (fold change), it represents the relative change in atretic follicles compared to normal follicles

**Fig. 4:**
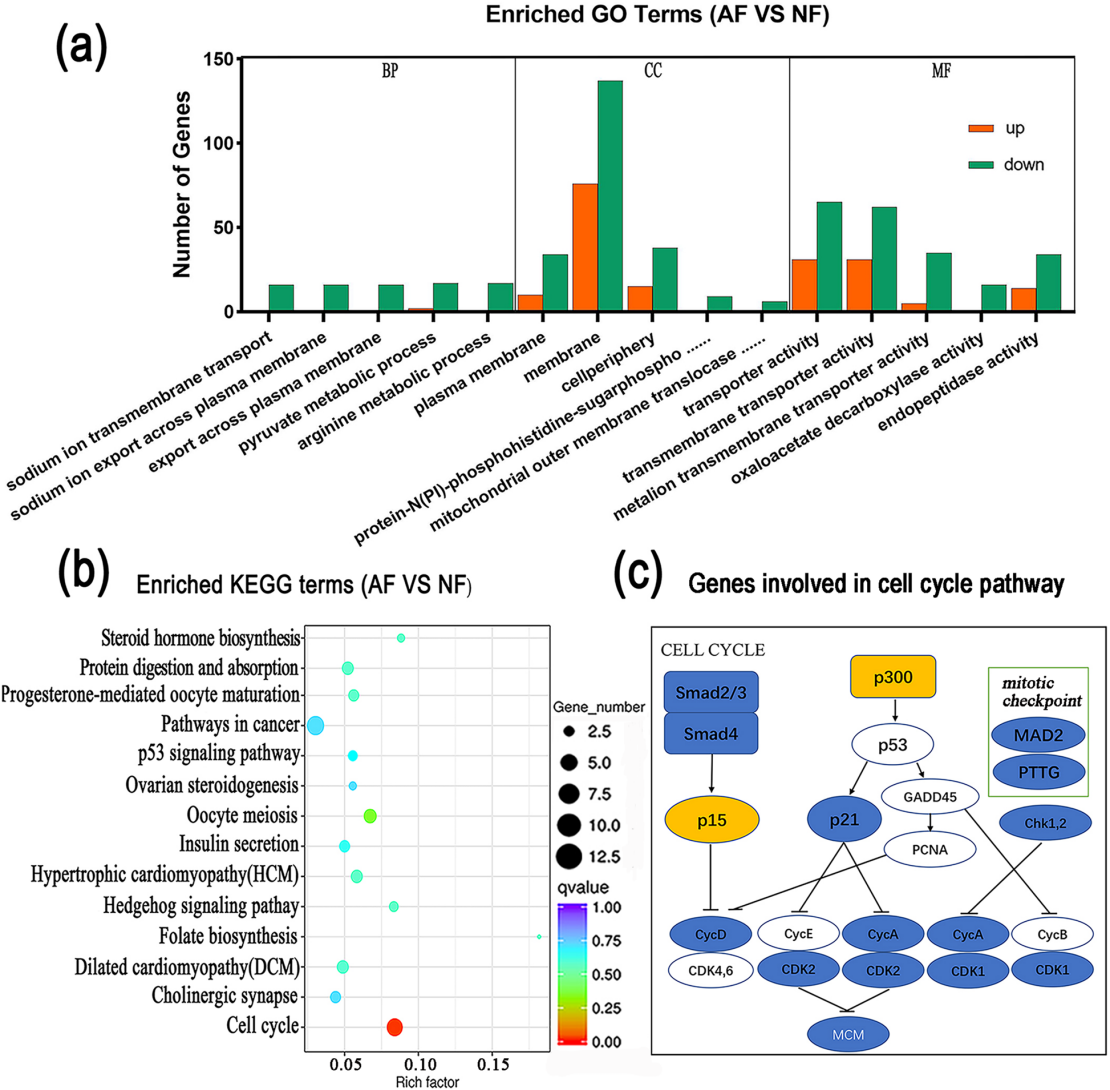
**a** GO analysis of downregulated or upregulated DEGs. BP: biological processes; CC: cellular components; MF: molecular functions. **b** The KEGG analysis of DEGs. **c** The DEGs involved in the cell cycle pathway. Yellow color represents upregulation and blue color represents downregulation in atretic follicles

The GO enrichment analysis was used for the functional analysis of DEGs. Among biological processes, DEGs were over-represented in the sodium ion transmembrane transport (GO: 0035725), pyruvate metabolic process (GO: 0006090), and arginine metabolic process (GO: 0006525). Regarding cellular components, DEGs were over-represented in the plasma membrane (GO: 0005886), cell periphery (GO: 0071944), andprotein-N(PI)-phosphohistidine-sugar phosphotransferase complex (GO: 0009357). In terms of the molecular functions, DEGs were over-represented in transmembrane transporter activity (GO: 0022857), oxaloacetate decarboxylase activity (GO: 0008948), and peptidase activity (GO: 0008233) (Supplementary Table S6, Fig. 4a). Based on the KEGG enrichment analysis, the DEGs were over-represented only in the cell cycle pathway (*P* < 0.05) in which Smad 2–3, Smad 4, cyclin D, cyclin A, cyclin-dependent kinase (*CDK1*, *CDK2*), mini-chromosome maintenance (*MCM3*), Myelin transcription factor 1 (*Myt1*), Serine/threonine-protein kinase (*chk1*, *chk 2*), mitotic spindle assembly checkpoint protein (*mad2*), and Securin (*PTTG*) were downregulated, while the ECREB-binding protein (*p300*) and cyclin-dependent kinase inhibitor (*Ink4b*) were upregulated (Supplementary Table S7, Fig. 4b&c).

**Fig. 5:**
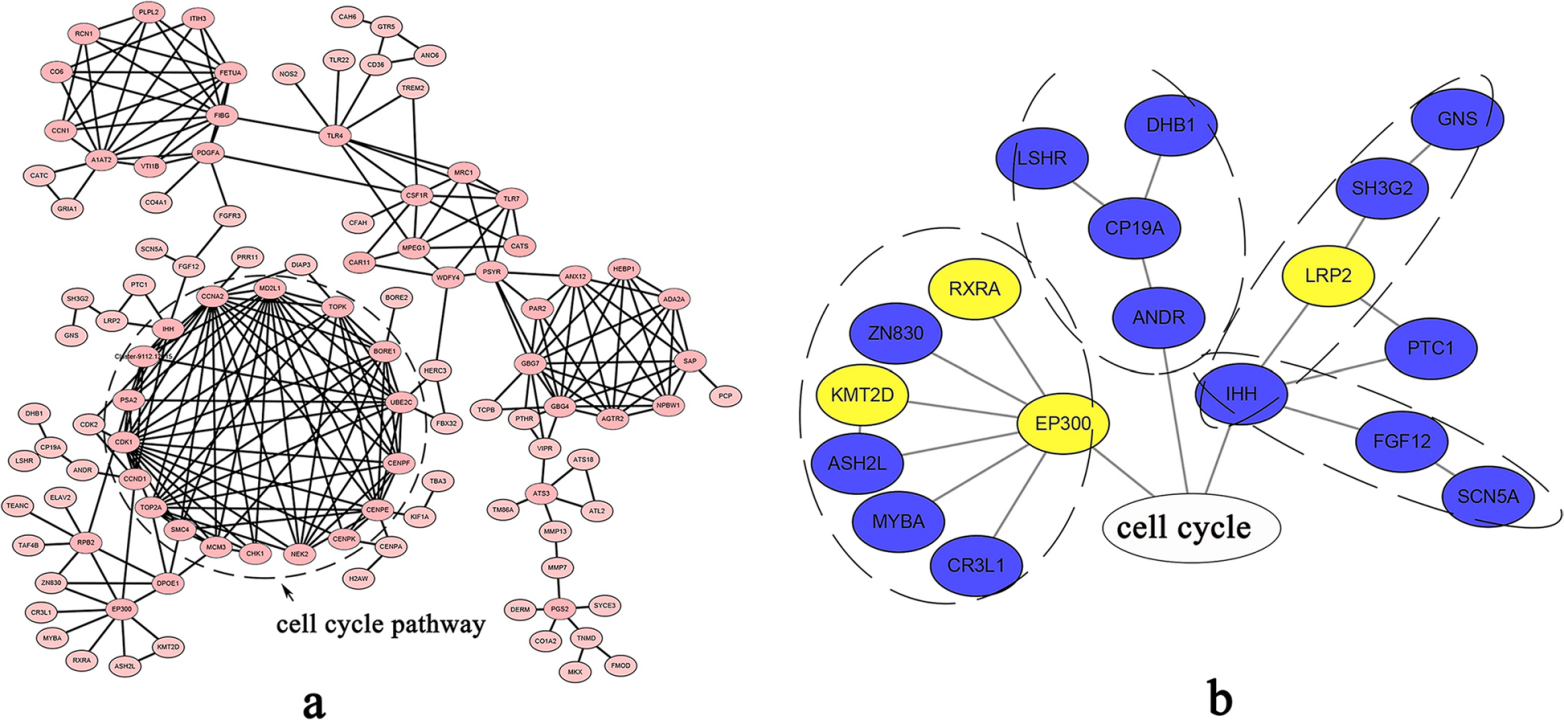
**a** The Protein−Protein Interaction Network (PPI) analysis of all DEGs. **b** Genes that interact with the cell cycle pathway. The yellow color represents upregulation, and the blue color represents downregulation in the atretic follicles

The Protein−Protein Interaction Network (PPI) analysis was performed to further explore the key genes that regulate the occurrence of follicular atresia (Fig. 5a). In the PPI based on the transcriptome data, cyclic AMP-responsive element-binding protein 3-like protein 1 (*CR3L1*), Myb-related protein A (*MYBA*), Set1/Ash2 histone methyltransferase complex subunit (*ASH2L*), histone-lysine N-methyltransferase 2D (*KMT2D*), zinc finger protein 830 (*ZN830*), and retinoic acid receptor RXR-alpha (*RXRA*), which are involved in the regulation of cell cycle, upregulated the EP300 in atretic follicles. The downregulated lutropin-choriogonadotropin hormone receptor (*LSHR*), estradiol 17-beta-dehydrogenase 1 (*DHB1*), aromatase (*CP19A*), androgen receptor (*ANDR*), N-acetylglucosamine-6-sulfatase (*GNS*), endophilin-A1 (*SH3G2*), protein-patched homolog 1 (*PTC1*), sodium channel protein type 5 subunit alpha (*SCN5A*), and FGF12, and the upregulated low-density lipoprotein receptor-related protein 2 (*LRP2*) inhibited cell cycle in atretic follicles (Fig. 5b).

### Proteome analysis

**Fig. 6:**
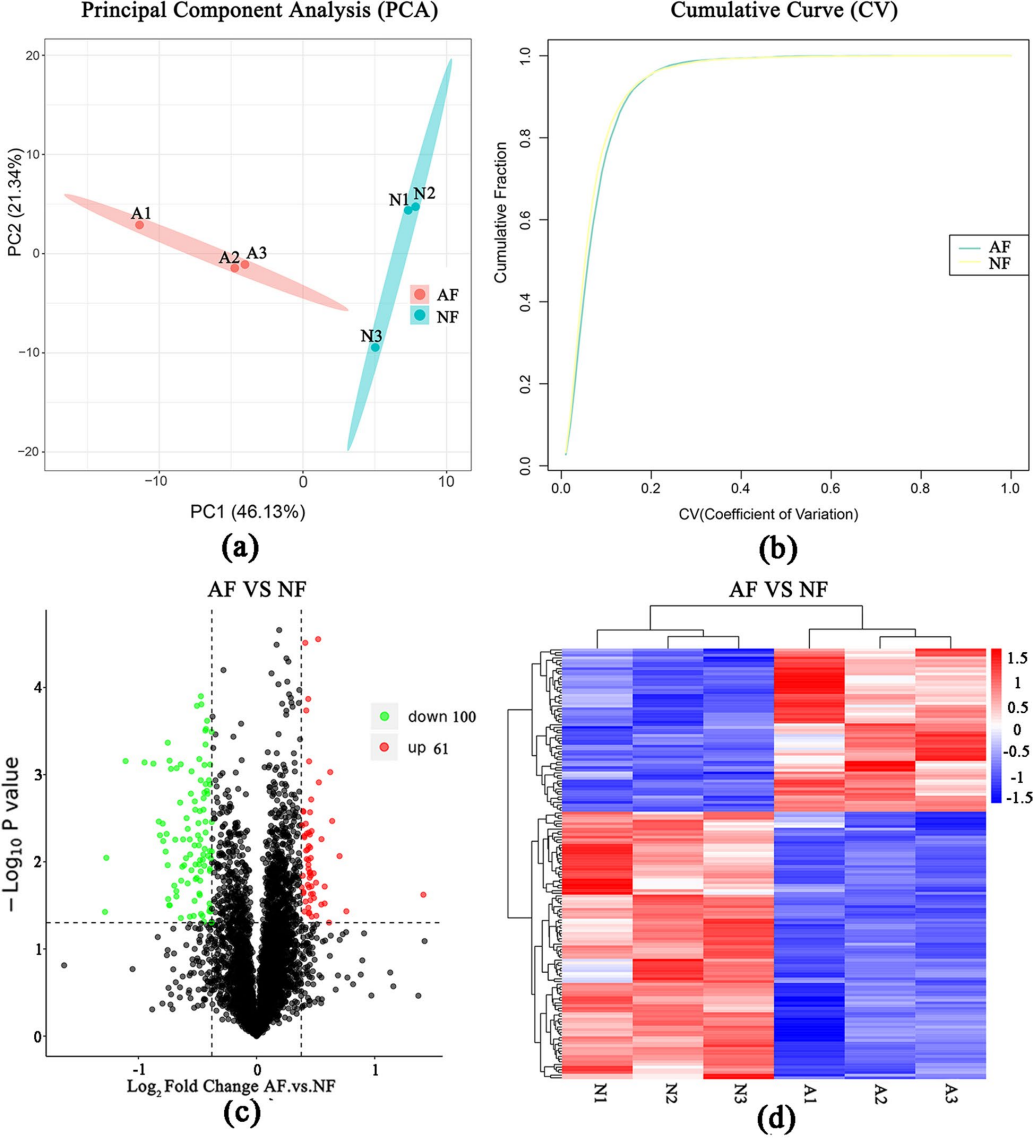
**a** Principal component analysis of DEPs in normal and atretic follicles. **b** Cumulative diagram of the values of coefficient of variance (CV) of all proteins in the corresponding sample. **c** Volcano plot of the differentially expressed proteins (DEPs) in the AF vs NF. AF: atretic follicles; NF: normal follicles. **d** The heatmap of hierarchical clustering of DEGs between the atretic and normal follicles

Mass spectrometry produced a total of 604,048 spectra, 61,114 matched spectra, 30,333 peptides, 4657 identified proteins, and 4654 quantifiable proteins (Supplementary Table S8). The length of most peptides varied between eight and 16 amino acids, protein coverage more than 10% was 54.07%, and the protein mass was mainly distributed in the range of 20 kDa–30 kDa and > 100 kDa (Supplementary Fig. S2). The principal component analysis (PCA) indicated a significant difference in protein expression between AF and NF groups (Fig. 6a). A low coefficient variance (CV) of 0.2 indicated good repeatability of each proteomic library (Fig. 6b).

A total of 161 DEPs was obtained, among which 61 were upregulated and 100 were downregulated in AF (Fig. 6c & d, Supplementary Table S9). The upregulated protein with the highest change was the *MMP*3 (log2FC = 1.41), responsible for proteolysis. The downregulated protein with the highest change was the U3 small nucleolar RNA-associated protein 18 homolog (UTP18, log2FC = − 1.28), responsible for protein processing (Table 2). The three proteins: vitellogenin-2-like (down), transgelin isoform X1 (up), and low-density lipoprotein receptor-related protein 1 isoform X1 (down) showed the highest expression change among DEPs (Supplementary Table S9).

**Table 2:** The top 10 upregulated or downregulated proteins in goose AF vs NF

Protein ID^**a**^	Gene	Gene full name	C (%)^**b**^	Peptides^**c**^	Log2FC^**d**^	UP/DOWN
Cluster-9112.25489;orf2	*MMP3*	stromelysin-1-like	9	1	1.41	up
Cluster-9112.14658;orf2	*HBAD*	hemoglobin alpha D subunit	10	1	0.75	up
Cluster-9112.10601;orf1	*HSPB7*	heat shock protein beta-7	10	1	0.70	up
Cluster-9112.22775;orf1	*MLEC*	myosin light chain 3	16	2	0.63	up
Cluster-9112.23899;orf1	*PRC2A*	Protein PRRC2A	9	1	0.62	up
Cluster-9112.6017;orf1	*LYG*	lysozyme g	25	4	0.61	up
Cluster-9112.9523;orf1	*TPPC1*	trafficking protein particle complex subunit 1	13	2	0.58	up
Cluster-9112.12518;orf1	*IF2B*	Eukaryotic translation initiation factor 2 subunit 2, partial	14	1	0.58	up
Cluster-9112.19651;orf1	*IKBE*	NF-kappa-B inhibitor epsilon	3	1	0.55	up
Cluster-9112.13569;orf1	*MMP9*	matrix metalloproteinase-9-like, partial	38	19	0.55	up
Cluster-9112.10020;orf1	*UTP18*	U3 small nucleolar RNA-associated protein 18 homolog	2	1	−1.28	down
Cluster-9112.11244;	*AAKB1*	minus strand	1	1	−1.27	down
Cluster-9112.4474;orf1	*GNMT*	glycine N-methyltransferase	3	1	−1.11	down
Cluster-9112.11859;orf1	*CCNT1*	cyclin-T1	2	1	−0.95	down
Cluster-9112.13068;orf1	*PLIN3*	perilipin-3 isoform X1	21	5	−0.87	down
Cluster-9112.12939;orf1	*NOP53*	hypothetical protein AV530_003974	3	1	−0.83	down
Cluster-9112.8116;orf1	*A1AT*	alpha-1-antitrypsin-like	3	1	−0.82	down
Cluster-9112.15043;orf2	*POL*	hypothetical protein DUI87_09561	18	13	−0.79	down
Cluster-9112.13866;orf1	*IPO13*	Importin-13, partial	4	3	−0.79	down
Cluster-9112.11626;orf1	*NICA*	nicastrin	8	4	−0.77	down

^a^The number of proteins obtained by mass spectrometry and annotation corresponding to the transcript database

^b^Sequence coverage (%), the ratio of the number of amino acids in every peptide that matches with the mass spectrum in the total number of amino acids in the protein sequence

^c^Number of matching peptides

^d^Relative change (x-fold) in atretic follicles compared to normal follicles

**Fig. 7:**
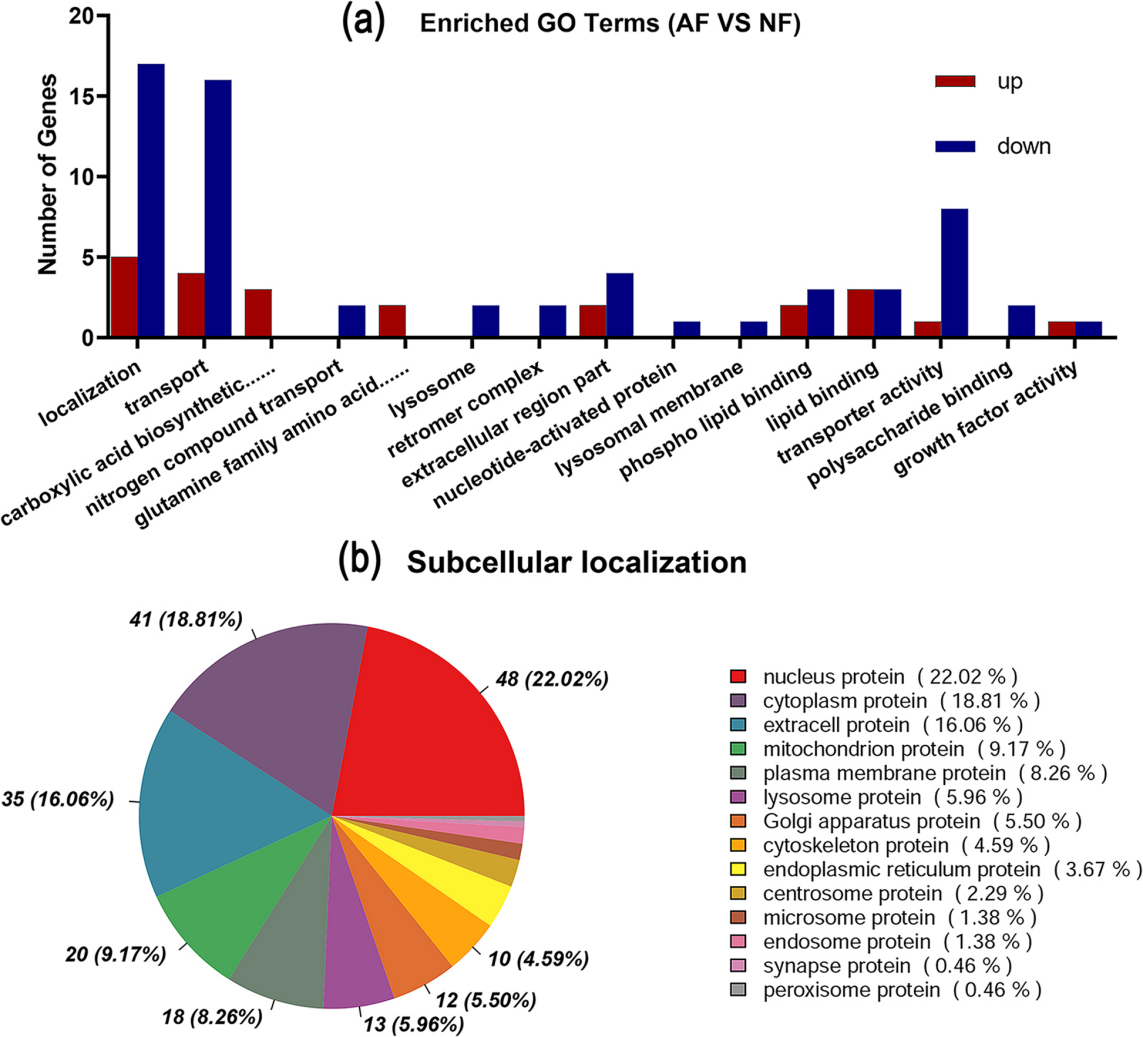
**a** GO analysis of the differentially expressed proteins (DEPs). BP: biological processes; CC: cellular components; MF: molecular functions. **b** The subcellular localization of DEPs

Among the biological processes, DEPs were over-represented in localization (GO: 0051179), transport (GO: 0006810), and transport of organic substances (GO: 0071702). Regarding the cellular components, DEPs were over-represented in the lysosome (GO: 0005764), retromer complex (GO: 0030904), and the extracellular region (GO: 0044421). In the case of molecular function, the DEPs were over-represented in phospholipid binding (GO: 0005543), lipid binding (GO: 0008289), and transporter activity (GO: 0005215, Fig. 7A, Supplementary Table S10). The subcellular localization of DEPs was mainly in the nuclear protein (22.02%), cytoplasm protein (18.81%), and extracellular protein (16.06%, Fig. 7b).

**Fig. 8:**
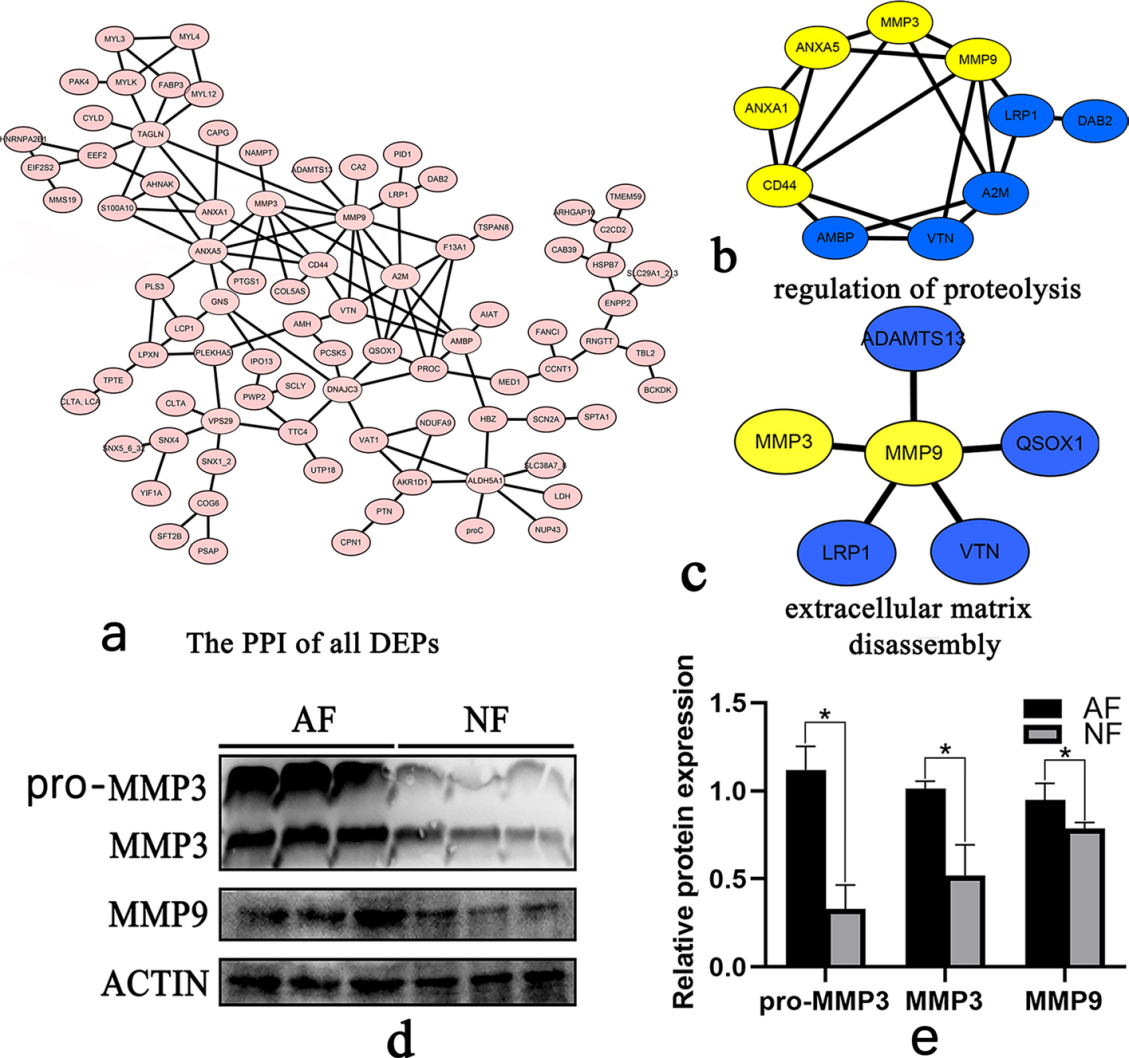
**a** The Protein−Protein Interaction Network (PPI) of all DEPs. **b** The PPI of proteins that were involved in the regulation of proteolysis. **c** The PPI of proteins involved in the degradation of the extracellular matrix. Yellow color represents upregulation and blue represents downregulation in the atretic follicle. **d** Western blot analysis of normal and atretic follicles. From left to right, lanes 1–3 are the three repeats of atreated follicles, and lanes 4–6 are the three repeats of normal follicles. Complete gels are presented in Supplementary Fig. S3. **e** The relative protein expression level of *MMP3* and *MMP9*

The PPI of proteome showed that the upregulation of MMP3, MMP9, CD44, annexin A1 (*ANXA1*), *ANXA5*, and the downregulation of low-density lipoprotein receptor-related protein 1 (*LRP1*), disabled homolog 2 (*DAB2*), alpha-2-macroglobulin (*A2M*), vitronectin (*VTN*), and *AMBP* were involved in proteolysis (Fig. 8a & b). The upregulation of *MMP3* and *MMP9*, along with the downregulation of A disintegrin and metalloproteinase with thrombospondin motifs 13 (*ADAMTS13*), sulfhydryl oxidase 1 (*QSOX1*), *VTN*, and *LRP1* were involved in the disassembly of the extracellular matrix. The upregulated expression of *MMP3* and *MMP9* played a key role in regulating follicle atresia through the promotion of protein degradation (Fig. 8c). To test the accuracy of mass spectrometry, western blotting of *MMP3* and *MMP9* was performed, and the results revealed high expression of *MMP3* and *MMP9* in atretic follicles (Fig. 8d & e).

### Integrated analysis of transcriptome and proteome

**Fig. 9:**
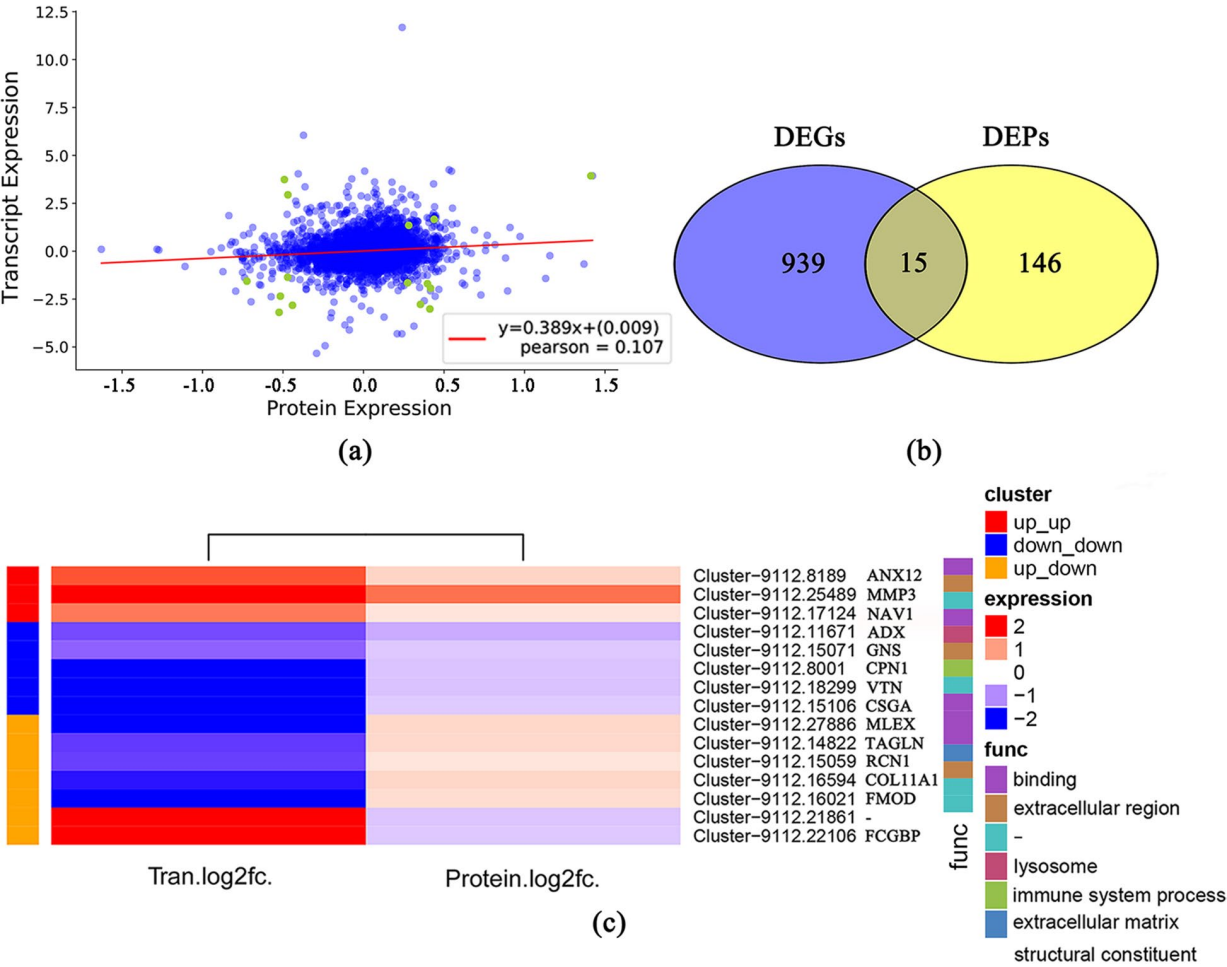
**a** Correlation analysis between protein expression and mRNA expression. **b** Venn diagram of differentially expressed genes and proteins between the atretic and normal follicles. **c** Functional cluster analysis of genes expressed in both transcriptome and proteome. Tran.log2fc represents the log2fc of the gene in the transcriptome, and Protein.log2fc represents the log2fc of the gene in the proteome

A weak correlation between the mRNA and protein expression was observed according to Pearson’s correlation coefficient (0.107) (Fig. 9a, Supplementary Table S11). Fifteen DEGs were identified both in proteome and transcriptome and eight showed the same expression trend (Fig. 9b, Table 3). Among them *MMP3*, neuron navigator 1 (*NAV1*), and *ANXA1* were upregulated, while adrenodoxin-like (*ADX*), glucosamine (N-acetyl)-6-sulfatase (*GNS*), carboxypeptidase N subunit 1 (*CPN1*), C-factor (CSGA), and *VTN* were downregulated. The GO analysis indicated that the DEGs in both proteome and transcriptome were mainly enriched in the binding and extracellular region (Fig. 9c).

**Table 3:** Differentially expressed genes at both mRNA and protein levels in the atretic and normal follicles in geese

Unigenes ID^**a**^	Symbol	description	mRNA^**b**^	Protein^**c**^
Cluster-9112.25489	*MMP3*	*Anser cygnoides* domesticus stromelysin-1-like (LOC106034136)	UP	UP
Cluster-9112.17124	*NAV1*	PREDICTED: *Anas platyrhynchos* neuron navigator 1	UP	UP
Cluster-9112.8189	*ANXA1*	*Anser cygnoides* domesticus annexin A1	UP	UP
Cluster-9112.11671	*ADX*	adrenodoxin-like (LOC106045559), transcript variant X3	DOWN	DOWN
Cluster-9112.15071	*GNS*	*Anser cygnoides* domesticus glucosamine (N-acetyl)-6-sulfatase	DOWN	DOWN
Cluster-9112.8001	*CPN1*	*Anas platyrhynchos* carboxypeptidase N subunit 1, transcript variant X2,	DOWN	DOWN
Cluster-9112.15106	*CSGA*	*Anas platyrhynchos* C-factor (LOC101798590)	DOWN	DOWN
Cluster-9112.18299	*VTN*	*Anas platyrhynchos* vitronectin (VTN), transcript variant X1	DOWN	DOWN
Cluster-9112.27886	*MLEX*	*Anser cygnoides* domesticus myosin, light chain 4, alkali; atrial, embryonic (MYL4), transcript variant X3	DOWN	UP
Cluster-9112.14822	*TAGLN*	*Gallus gallus* transgelin (TAGLN), mRNA && M83105.1 Chicken SM22 mRNA, complete cds	DOWN	UP
Cluster-9112.16594	*COL11A1*	*Anas platyrhynchos* collagen type XI alpha 1 chain (COL11A1), transcript variant X2	DOWN	UP
Cluster-9112.16021	*FMOD*	*Anas platyrhynchos* fibromodulin (FMOD)	DOWN	UP
Cluster-9112.15059	*RCN1*	*Anser cygnoides* domesticus reticulocalbin 1, EF-hand calcium binding domain (RCN1)	DOWN	UP
Cluster-9112.22106	*FCGBP*	*Nipponia nippon* IgGFc-binding protein-like (LOC104012603),	UP	DOWN
Cluster-9112.21861	–	*Anser cygnoides* domesticus uncharacterized (LOC106034650),	UP	DOWN

^a^The unique identification number of a gene in the sequencing file

^b^The upregulation or downregulation of genes in transcriptome of atretic follicles compared to normal follicles

^c^The upregulation or downregulation of genes in the proteome of atretic follicles compared to normal follicles

### qRT-PCR analysis of the candidate genes

**Fig. 10:**
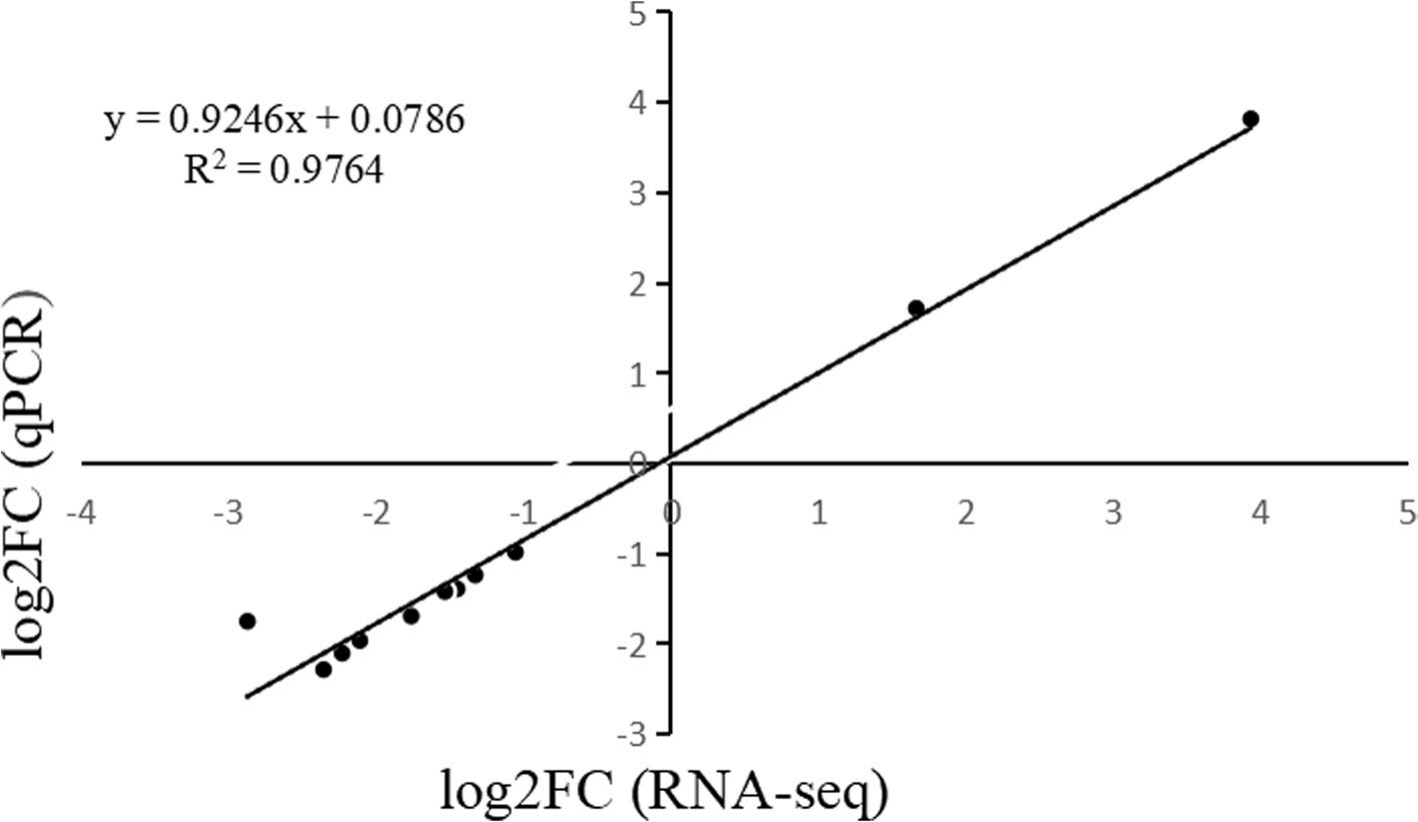
Correlation analysis between the log2FC (RNA-Seq) and log2FC (qPCR). The genes verified by qPCR included *PDGFA*, *FGF12*, *CHK1*, *CCND1*, *ANX12*, *Smad4*, *CCNA2*, *MCM3*, *Smad 2/3*, *MAD2*, *CDK1* and *MMP3*

According to the transcriptome, twelve candidate genes were selected and verified. *CHK1, MCM3, CCNA2, MAD2, CDK1*, and *CCND1* at the nodes and played important role in cell cycle pathway. *Smad2–3* and *Smad4* at the transcriptional factors of *TGFβ* for transmembrane signal transduction. *ANXA1* and *MMP3* that differentially expressed in both transcriptome and proteome were chosen for additional quantitative determination with qRT-PCR. The expression level of the selected genes followed a similar trend (R^2^ = 0.9764) in the relative expression levels between the log2FC (RNA-Seq) and log2FC (qPCR), indicating the reliability of our transcriptome data (Fig. 10).

## Discussion

Reproductive hormones play an important regulatory role in follicular development. The FSH and LH promote follicular maturation and ovulation and inhibit the apoptosis of granulosa cells in human [13]. Ilha et al. suggested that the activation of *LIF-STAT3* in follicular granulosa cells resulted in the decreased level of FSH and causes follicle atresia in cattle [14]. The FSH and LH are glycoproteins that are secreted by the pituitary gland and need to be transported through the blood to the target site to be effective. A study by Feranil et al. demonstrated that the distribution of peripheral vasculature in atretic follicles was lesser than that of normal follicles in swamp buffalo [15], which possibly led to lower levels of FSH and LH. The in vitro inhibition of angiogenesis could directly induce the apoptosis of granulosa cells and thus caused follicular atresia in rats [16]. Lower levels of FSH and LH were also detected in AF of Yangzhou geese in this study, which demonstrated that steroid hormones were involved in follicle atresia. The *PDGFA*, which regulated cell growth and angiogenesis by binding to its receptor, could increase follicle size and enhance the transition from primordial to primary follicles in the in vitro culture of sheep follicles [17], was downregulated in AF in this study. This suggested that decreased angiogenesis was associated with follicle atresia.

Granulosa cells represented the first site of the initiation of apoptosis in rat atretic follicles [9], and follicular growth or atresia were regulated by the survival or death of granulosa cells in mammalian ovaries [12]. All sodium ion transmembrane transport-related genes were downregulated in the atretic follicles, especially in the plasma membrane, which caused the efflux of sodium ions and thus initiated the activation and shrinkage of apoptotic cells [18, 19], which may explain the obvious collapse in the atretic follicles of Yangzhou geese in this study. The caspase recruitment domain (*CARD*) 9 and 11 showed a significant increase in atretic follicles at the transcriptional level [20], while the mitochondrial proteins *SmaC* and *HtrA2*, which promoted cytochrome C-dependent caspase activation by the inhibition of *IAP*, were upregulated in atretic follicles [21]. These studies demonstrated that apoptotic related genes were highly expressed to initiate the apoptosis of atretic follicles, which also need transcriptome and proteome levels changes to complete granulose cell apoptosis and follicle atresia.

Huet et al. believed that changes in ECM components could affect the apoptosis of follicular cells in sheep [22]. Matrix metalloproteinase (*MMP*) participates in the disassembly of the extracellular matrix, and the upregulated proteins *MMP3* and *MMP9* could be involved in the collapse of the granulosa cell layer. As a secretion protein, *MMP* acted on growth factors and growth factor-binding proteins to affect various cellular functions, including cell invasion, cell migration, apoptosis, and angiogenesis [23]. In the follicles of humans and rats, *MMP* promoted ovulation by destroying the follicle wall and the surrounding matrix through the stimulation of LH and HCG [24]. After ovulation, the ovaries of poultry leave postovulatory follicles, which gradually degenerate and disappeared. Hrabia et al. reported that the increased activity of *MMP2* and *MMP9* promoted follicle regression in chicken postovulatory follicles [25]. Zhu et al. also reported that the protein expression of *MMP9* increased with the degree of regression of postovulatory follicles [26]. The upregulation of *MMP3* and *MMP9* detected in this study in the atretic follicles of Yangzhou geese also suggested that these two genes were involved in the apoptosis of granulosa cells in AF, and might also promote the degeneration and disappearance of atretic follicles.

A complex composed of *CDK* and cyclin synergistically promoted the cell cycle, while the downregulation of *CDK* (*CDK1* and *CDK2*) and cyclin (*CyCD* and *CyCA*) caused a decrease in the cell division activity in atretic follicles. The downregulation of *Smad 2–3* and *Smad 4* resulted in the interruption of cell growth or transformation. Tomic et al. demonstrated that the decreased expression of Smad3 resulted in the downregulation of *CyCD2* and increased follicular atresia in mice [27]. On the other hand, the increased expression of *p300* and *Ink4b* caused a high level of acetylation of *p53* and resulted in the increased expression of *p21*, which then bound to *CDK* and *CyC* to interrupt the cell cycle process. Regan et al. demonstrated that high *LSHR* was associated with a higher number of follicles, reduced apoptosis, and a higher ovulation rate in merino sheep [28]. A study reported the reduction of *ANDR* in atretic porcine follicles [29], while another reported that the mRNA of *ANDR* could be regulated by FSH [30]. FGF12, a growth factor, could promote the migration of endothelial cells, proliferation of smooth muscle cells, formation of new blood vessels, and the repair of damaged endothelial cells, which could act intracellularly to inhibit apoptosis [31, 32]. The downregulation of *FGF12* could also be involved in the apoptosis of atretic follicles. The *bFGF*, *FGF10*, and *FGF18* have been reported to be closely associated with follicular development and atresia [4–6]. In this experiment, the PPI of DEPs suggested that the downregulated genes *LSHR*, *DHB1*, *CP19A*, *ANDR*, and *FGF12*, which participated in the cell cycle pathway, could be the key factors regulating follicular atresia in Yangzhou geese.

In this study, there was weak weak correlation between transcriptome and proteome (R2 = 0.107). Zhao et al. showed that the low quantitative correlation between the transcriptome and proteome in wild-type and *fads2*-deletion zebrafish, with R2 values of 0.012 and 0.076, respectively [33]. The low correlation between transcriptome and proteome results may be due to complex post-transcriptional regulation. Differentially expressed genes in both transcriptome and proteome mainly enriched in binding and extracellular region signal pathways. *ANXA1* was a calcium-dependent phospholipid-binding protein that is expressed in almost all organs [34]. It participated in membrane transport, exocytosis, the fusion of some membranes, signal transduction, cell proliferation, apoptosis, and ion channel formation [35]. Zhu et al. reported that *ANXA2* was involved in the angiogenesis of chicken ovarian follicles [36]. The downregulation of *VTN*, an extracellular matrix protein, was the intermediate cause of decreased adhesion between granulosa cell layers. *VTN* could be degraded by *MMPs* [37], and its content was positively correlated with the follicular size in bovine [38]. The decreased expression of *VTN* further suggested that it might be degraded in atretic follicle and caused the collapse of cell layer.

## Conclusions

In this experiment, normal and atretic follicles from geese were compared using integrated transcriptome and proteome analysis. The cell cycle is an important pathway for the regulation of follicular atresia. Sodium outflow and high expression of *MMP3* and *MMP9* were important causes of structural destruction and apoptosis of follicular cells. The genes *Smad2/3*, *Smad4*, *ANXA1*, *MMP3*, *ANXA1*, *CHK1*, *MCM3*, *CCNA2*, *MAD2*, *CDK1*, *FGF12*, and *CCND1*, which were involved in the cell cycle, could be candidate genes for the regulation of follicular atresia.

## Materials and methods

### Experimental animals and sample collection

Six egg-laying geese (provided by Jiangsu Changzhou Four Seasons Poultry Industry Co., Ltd.) were selected for slaughter after being anesthetized with sodium pentobarbital. The follicles which displayed typical morphology of atresia were separated as the atretic follicles (AF). Normal developing follicles (NF) with similar size of AF were separated and set as the control. One part of the separated follicles was immediately frozen in liquid nitrogen and then stored at − 80 °C for hormone determination. In each follicle development stage, the large white follicles were selected as the object for transcriptome and proteome. From the second part, follicle granulose cell layer was immediately separated and stored for, RNA and protein extraction. The remaining part was stored in a tissue-fixative solution (4% paraformaldehyde) for the paraffin section.

### ELISA and TUNEL assays

After 24 h of immersion in the tissue-fixative solution, the AF and NF were embedded in paraffin and sectioned into slices of 5 μm thickness. These slices were then subjected to TUNEL staining using the TUNEL Apoptosis Detection Kit (yeasen, China) according to the manufacturer’s instructions. The levels of FSH, LH, PRL, E2, and P4 hormones were measured using the Enzyme-Linked Immunosorbent assay (ELISA) according to the manufacturer’s instructions (Keshun tech, Shanghai, China). The microtiter plate was read at 450 nm using microtiter plater reader (SUNRISE, Astria). Twelve follicles in each of the NF and AF groups were used for the measurement of hormone levels. .

### De novo transcriptome analysis

The total RNA was extracted from each follicle. Three extracted samples were equally mixed to form one replicate, and three replicates represented nine samples that were submitted for analysis. Total RNA was extracted using the Animal Tissue RNA Extraction Kit (Ambion, USA). The purity of the extracted RNA was assessed using the RNA Nano 6000 Assay Kit (Bioanalyzer 2100 system, Agilent Technologies, CA, USA). Purification and reverse transcription of the mRNA were performed using the NEBNext Ultra RNA Library Prep Kit (Illumina, NEB, USA). The cDNA fragment of 370 ~ 420 bp was selected for PCR and repurified using the AMPure XP system (Beckman Coulter, Beverly, USA). The quantity and quality of the final library was assessed using the Qubit 2.0 Fluorometer (Thermo Fisher, USA) and Agilent Bioanalyzer 2100 system (Agilent, USA), respectively.

The mRNA library was sequenced using Illumina NovaSeq 6000. Clean reads were obtained from raw data by removing reads containing the adapter, ploy-N, and low-quality. De novo assembly was performed using the software Trinity [39], and each cluster was defined as a “Transcript”. The “UniGenes” were obtained by removing redundant data from the “Transcripts” using the software Corset [40]. The completeness of transcript assembly was assessed by the Benchmarking Universal Single-Copy Orthologs (BUSCO, http://busco.ezlab.org/).

Functional annotation of the UniGenes was performed using seven databases: NCBI non-redundant protein sequences (Nr), NCBI nucleotide sequences (Nt), Protein family (PFAM), eukaryotic ortholog groups (KOG), SwissProt, Kyoto Encyclopedia of Genes and Genomes (KEGG), and gene ontology (GO). To calculate the expression of the UniGenes, clean reads were mapped to the the reference database assembled by Trinity using the software RSEM [41]. The gene expression was quantified and normalized by the calculation method of “Reads per kilobase per million mapped reads” (RPKM) [42], and RPKM values > 0.3 represented that the gene was detected in follicle.

### Proteomic analysis

The total protein from each follicle was extracted using the acetone precipitation method. Protein samples were extracted from nine follicles each of normal and atretic follicles. Three samples were equallty mixed to form one replicate and three replicates were submitted for proteome analysis. The concentration and quality of the extracted protein were measured using the Bradford Protein Quantitative Kit (P0006, Beyotime, Shanghai, China) and 12% SDS-PAGE electrophoresis, respectively. After the protein samples were analyzed, digested, and desalinated, they were labeled using isobaric tags (iTRAQ) for relative and absolute quantification [43]. Briefly, the protein samples were digested with trypsin overnight, and the pH was adjusted to around 3.0 using formic acid (FA). The samples were then centrifuged at 12000 g for 5 min and the supernatant was collected for desalting through the C18 desalting column with elution buffer of 0.1% FA and 70% acetonitrile. Each sample was reconstituted with 20 μL of 1 M TEAB buffer (Sigma, USA) and iTRAQ Reagent-8 plex kit (Sigma, USA) for 2 h, following which, 100 μL of 50 mM Tris-HCl (pH = 8) was added to terminate the reaction. The labeled samples were fractionated using a C18 Nano-Trap column (4.6 × 250 mm, 5 μm, Thermo Fisher, USA) on a Rigol L3000 UHPLC system and monitored at 214 nm. Finally, all fractions were dried under vacuum and reconstituted in 0.1% FA.

The liquid chromatography-tandem mass spectrometry (LC-MS/MS) was performed on an EASY-nLCTM 1200 UHPLC system (Thermo Fisher, Massachusetts, USA) coupled to a Q Exactive™ HF-X mass spectrometer (Thermo Fisher, Massachusetts, USA) and operated in a data-dependent acquisition mode [44]. The fraction sample was injected into a C18 Nano-Trap column (4.5 cm × 75 μm, 3 μm, Thermo Fisher, USA). The peptides were separated on an analytical column (15 cm × 150 μm, 1.9 μm) using linear gradient elution for 60 min with a flow rate of 600 nL/min. The gradient elution included mobile phase A (0.1% FA) varying from 94 to 0% and mobile phase B (0.1% FA, 80% acetonitrile) varying from 6 to100%. A full MS scan ranging from 407 to 1500 m/z was conducted at 60,000 resolution with a target automatic gain control (AGC) value of 3 × 106 ions and a maximum ion injection time (IT) of 20 ms. The top 40 precursors of the highest abundance in the full scan were selected and fragmented using high-energy collisional dissociation (HCD). All MS/MS spectra were scanned using the following parameters: resolution = 15,000, AGC = 5 × 104 ions, maximum IT = 45 ms, dynamic exclusion duration = 20 s, normalized collision energy = 32%, and intensity threshold = 2.2 × 104.

The coding sequence (CDS) of the UniGenes from transcriptome was searched in the Nr and SwissProt protein databases. Once the CDS could not match to these two protein database, it would be predicted by the software ESTSCAN version 3.0.3. This combined database was the reference for the proteomic analysis. All spectra were searched against the transcriptome CDS prediction database using the software Proteome Discoverer 2.2 (PD 2.2, Thermo). The search parameters were set as follows: mass tolerance for precursor ion was 10 ppm, mass tolerance for production was 0.02 Da, the fixed modification was carbamidomethyl, dynamic modifications were Oxidation of methionine (M) and iTRAQ plex, N-Terminal modifications were acetylation and iTRAQ plex, and a maximum of two missed cleavage sites was allowed. To ensure the accuracy of the MS data analysis, the Peptide Spectrum Matches (PSMs), protein confidences, and false discovery rates (FDR) were set to > 99%, > 1.0, and ≤ 1.0%, respectively. The proteins were annotated using the program InterProscan against the GO, KEGG, IPR, and COG databases [45].

### qRT-PCR validation

Total RNA was extracted from the goose follicle and reverse transcribed for cDNA synthesis using Hifair® II 1st Strand cDNA Synthesis Kit (Yeasen, China) according to the manufacturer’s protocol. Real-time quantitative PCR (qRT-PCR) was performed using the TransStart Green qPCR SuperMix Kit (TAKARA, China) on an ABI 7500 system (Thermo, USA). The primers were designed using the online platform Primer 3.0 (https://bioinfo.ut.ee/primer3–0.4.0/) (Table 4). The conditions of qRT-PCR were: 95 °C for 5 min, followed by 40 cycles of denaturation at 95 °C for 10s and annealing and extension at 60 °C for 20 s. The expression level was calculated by using the 2^–ΔΔCT^ method.

**Table 4:** Primers used for the qRT-PCR validation

Gene	FORWARD PRIMER	RIGHT PRIMER	SIZE (BP)
smad2–3	GCTGGATGAGCTTGAGAAGG	ACGTGCGGTAATCCTTTACG	207
smad4	CAGATGCAGCAGCAAGCA	AGCCCTTGACGAAGCTGAG	290
ANX12	TATTGAGAACTGCCTGACTGCC	TGTCCCAAGGCCCTTCATTG	97
CHK1	GCTGGTGAAGAGGATGACGC	CTCCTGTCCGTGGTGGAGAT	144
MCM^3^	CCGCGACCAAGAAGACCATC	CCTTGTAGACCGAGAGGCCA	138
CCNA2	GCTGAGCTTGCACCTCTTCA	CCTCCCCAGTGAGAAGGATGT	134
MAD2	TTCACCCGCGTCCAGAAGTA	GCACTTGCAGAGCCACTCTT	111
CDK1	ATGGCCTGATGTGGAGTCCC	AAAGCAGATCAAGCCCATCTTCAT	113
CCND1	CGAGCCTGCCAAGAACAGAT	GTGTGCAGGAAAGGTCTGCT	118
FGF12	AGCTCGGATGTTTTCACACC	GCGTCCTTGTTTTTCTCCAA	258
MMP3	GGACATGATGAAGCAGCCCA	TCGTCCACATCAGCTTGCAG	144
PDGFA	GCCTTTGGCTTTATTGTGGA	GGTTTCCTTCACATCGGAGA	199

### Western blot analysis

Gel electrophoresis was performed using 12% SDS-PAGE, after which, the proteins were transferred onto PVDF membrane. The PVDF membranes were then incubated in 5% skim milk (diluted in TBST) for 2 h and then incubated in the diluted primary antibodies (rabbit anti-*MMP3* with 1:1000 dilution, YT4465, ImmunoWay, Jiangsu, China; anti-*MMP9* with 1:1000 dilution, YT1892, ImmunoWay, Jiangsu, China; anti-*β-actin* with 1:10000 dilution, ABclonal, Wuhan, China) at 4 °C overnight. After washing by tris-buffered saline three times, the PVDF membrane was incubated with HRP-labeled goat anti-rabbit IgG (1:30,000, BIOMIKY, Shanghai, China) for 60 min. Protein blots were visualized using chemiluminescence imaging system 398 (UVitec, Cambridge, United Kingdom) and quantified using Image J software (National Institutes of Health, USA).

### Statistical and bioinformatics analysis

The differences in expression of genes and proteins between AF and NF were analyzed using the T-test in the statistical program R. *P* values were corrected using Benjamini and Hochberg. The genes with p_adj_ of ≤0.05 and fold change (FC) of > 2 were considered differentially expressed (DEGs). The proteins with p_adj_ of ≤0.05 and FC of > 1.3 were defined as differentially expressed proteins (DEPs). The relative mRNA expression through qRT-PCR was analyzed using student T-test in the statistical software SPSS version 22.0 (SPSS, Chicago, IL, USA). The histogram was made using the software GraphPad Prism version 8.0 (GraphPad, San Diego, USA). The GO and KEGG enrichment analyses were performed using the software GOseq and KOBAS [46, 47]. Protein-protein interaction (PPI) was predicted and visually edited using the software STRING DB and Cytoscape, respectively, with String database (http://string-db.org/) as the reference [48].

## Supplementary Information

**Additional file 1: Fig. S1.** Principal component analysis of DEGs in normal and atretic follicles.

**Additional file 2: Fig. S2.** (a) The quality control of mass spectrometry, including peptide length, (b) precursor ion tolerance, (c) protein mass distribution, and (d) protein coverage.

**Additional file 3: Fig. S3.** (a) Western blotting of MMP3 protein in normal and atretic follicles. The remaining three bands, also normal follicles, were cropped out of the manuscript picture. (b) Western blotting of MMP9 protein in normal and atretic follicles. (c) Western blotting of ACTIN protein. AF-1, AF-2 and AF-3 represent the three repeats of atreated follicles, while NF-1, NF-2 and NF-3 represent the three repeats of normal follicles. The red area is the cropped area in the manuscript. The PVDF membrane was cut before incubation with antibodies.

**Additional file 4: Table S1.** Summary of transcriptome sequencing quality.

**Additional file 5: Table S2.** Summary of Transcripts and Unigene.

**Additional file 6: Table S3.** Summary of the functional annotation of assembled unigenes.

**Additional file 7: Table S4.** The FPKM value of all genes in the goose follicles.

**Additional file 8: Table S5.** The differentially expressed genes between normal and atretic follicles.

**Additional file 9: Table S6.** GO enrichment of differentially expressed genes between normal and atretic follicles (molecular_function).

**Additional file 10: Table S7.** KEGG enrichment of differentially expressed genes between normal and atretic follicles.

**Additional file 11: Table S8.** The expression of all proteins in the goose follicles.

**Additional file 12: Table S9.** The differentially expressed proteins between normal and atretic follicles.

**Additional file 13: Table S10.** GO enrichment of differentially expressed proteins between normal and atretic follicles (*p* < 0.05).

**Additional file 14: Table S11.** The expressions of mRNAs and proteins that corresponds to each other in the goose follicles.

## Data Availability

The mass spectrometry proteomics data have been deposited to the ProteomeXchange Consortium (http://proteomecentral.proteomexchange.org) via the iProX partner repository with the dataset identifier PXD028836. ‍The transcriptomics datasets generated during the current study are available in NCBI SRA (PRJNA767219, https://www.ncbi.nlm.nih.gov/bioproject/PRJNA767219).

## Abbreviations

NF: Normal follicles
AF: Atretic follicles
ELISA: Enzyme linked immunosorbent assay
Tunel: TDT-mediated dUTP Nick-end Labeling
qRT-PCR: Quantitative real-time PCR
RNA-seq: RNA-sequencing
iTRAQ: isobaric tags for relative and absolute quantification
PCA: Principal component analysis
PPI: Protein−Protein Interaction Network
