# Supplementary figures and images for: Integrated transcriptome and proteome revealed that the declined expression of cell cycle-related genes associated with follicular atresia in geese
Source: BMC Genomics. 2023 Jan 16;24:24. doi: 10.1186/s12864-022-09088-1 (PMC9843891; doi:10.1186/s12864-022-09088-1)

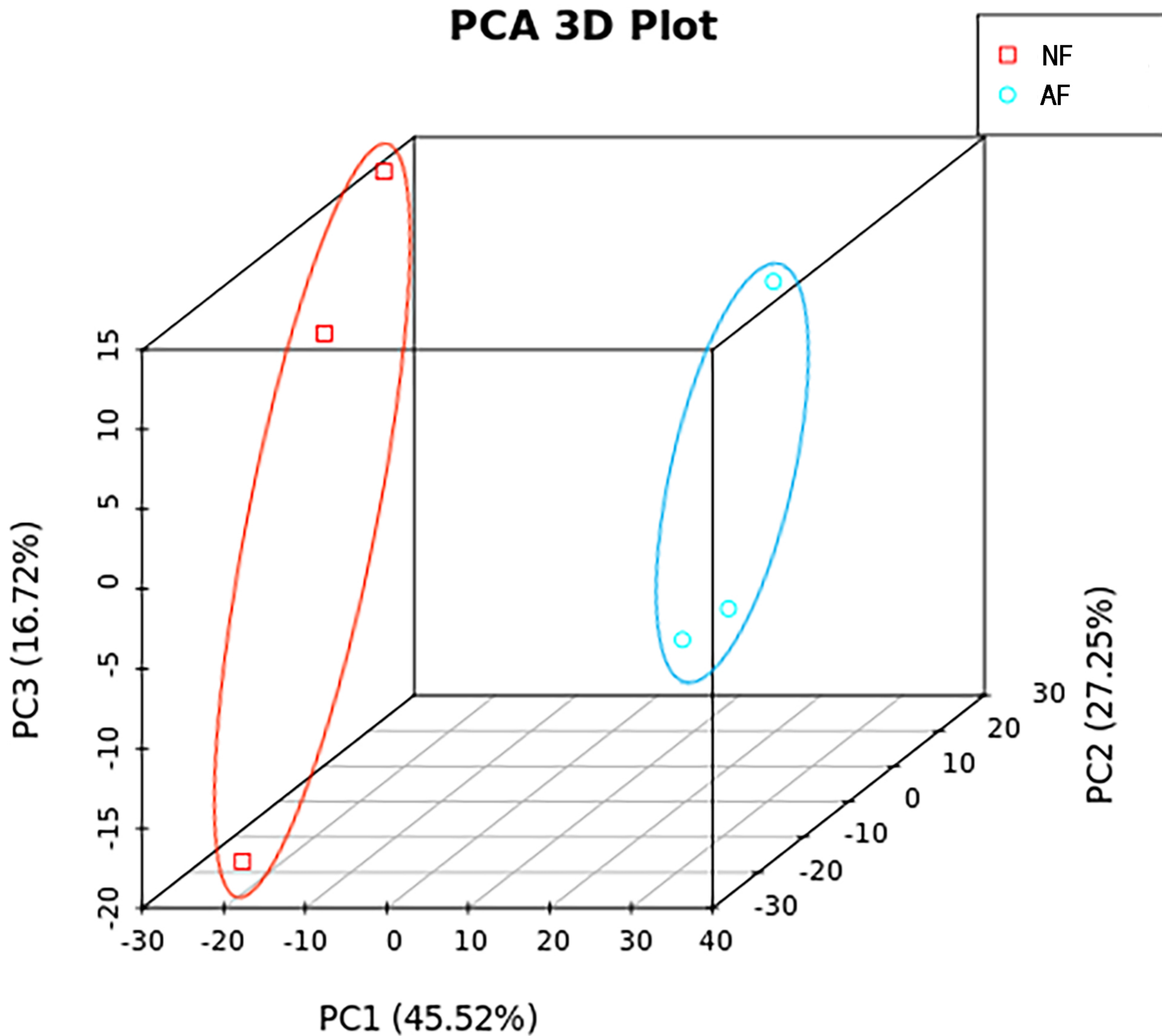

Figure S1. Principal component analysis of DEGs in normal and atretic follicles.

Supplement: Supplementary file 1 — Additional file 1: Fig. S1. Principal component analysis of DEGs in normal and atretic follicles. [file 12864_2022_9088_MOESM1_ESM.pdf]
