# Supplementary material for: Integrated transcriptome and proteome revealed that the declined expression of cell cycle-related genes associated with follicular atresia in geese
Source: BMC Genomics. 2023 Jan 16;24:24. doi: 10.1186/s12864-022-09088-1 (PMC9843891; doi:10.1186/s12864-022-09088-1)
Supplement: Supplementary file 2 — Additional file 2: Fig. S2. (a) The quality control of mass spectrometry, including peptide length, (b) precursor ion tolerance, (c) protein mass distribution, and (d) protein coverage. [file 12864_2022_9088_MOESM2_ESM.pdf]

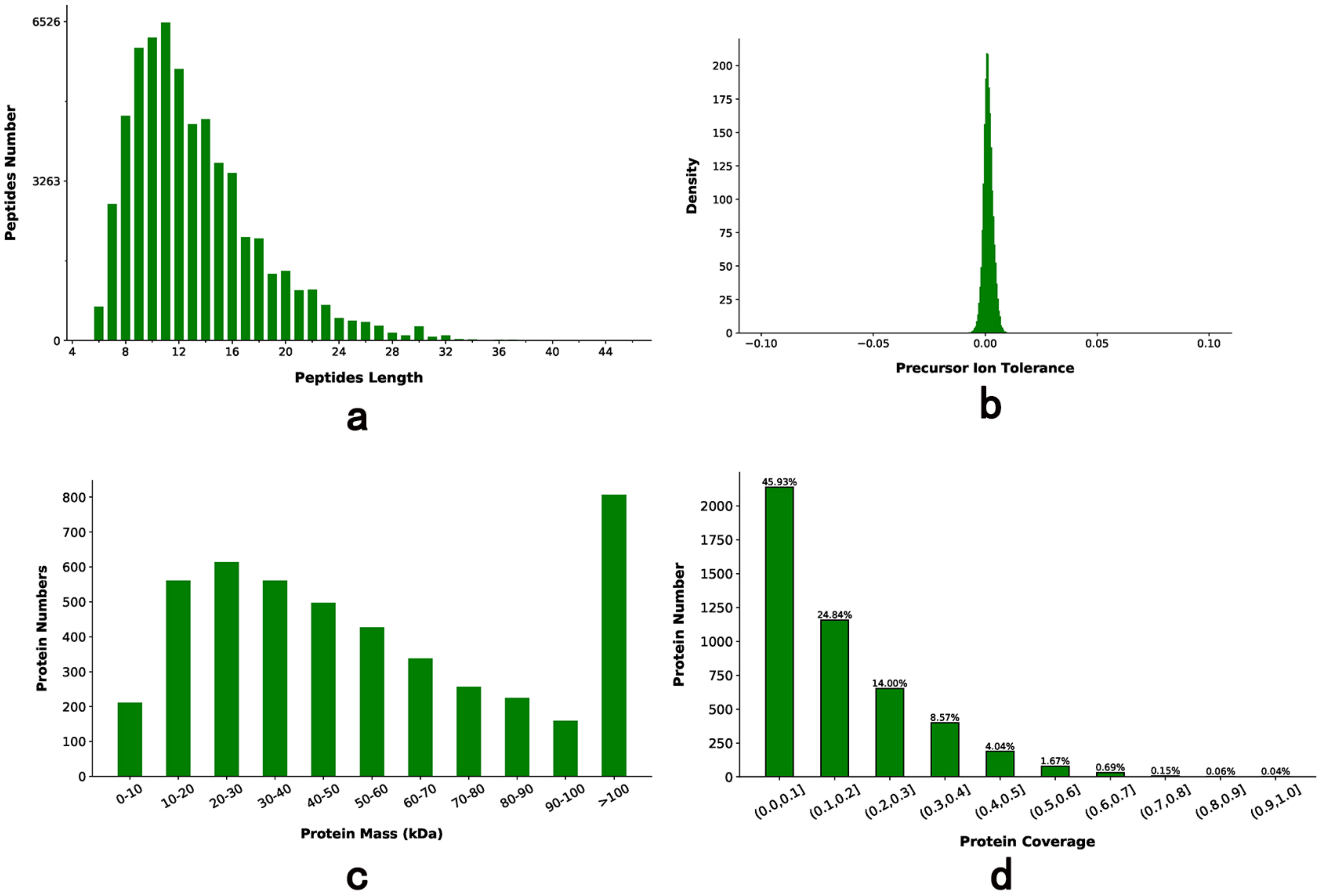


Figure S21. (a) The quality control of mass spectrometry, including peptide length, (b) precursor ion tolerance, (c) protein mass distribution , and (d) protein coverage.
