# Supplementary material for: Integrated transcriptome and proteome revealed that the declined expression of cell cycle-related genes associated with follicular atresia in geese
Source: BMC Genomics. 2023 Jan 16;24:24. doi: 10.1186/s12864-022-09088-1 (PMC9843891; doi:10.1186/s12864-022-09088-1)

Figure S3. (a) Western blotting of MMP3 protein in normal and atretic follicles.The remaining three bands, also normal follicles, were cropped out of the manuscript picture. (b) Western blotting of MMP9 protein in normal and atretic follicles. (c) Western blotting of ACTIN protein. AF-1, AF-2 and AF-3 represent the three repeats of atreated follicles, while NF-1, NF-2 and NF-3 represent the three repeats of normal follicles .The red area is the cropped area in the manuscript. The PVDF membrane was cut before incubation with antibodies.

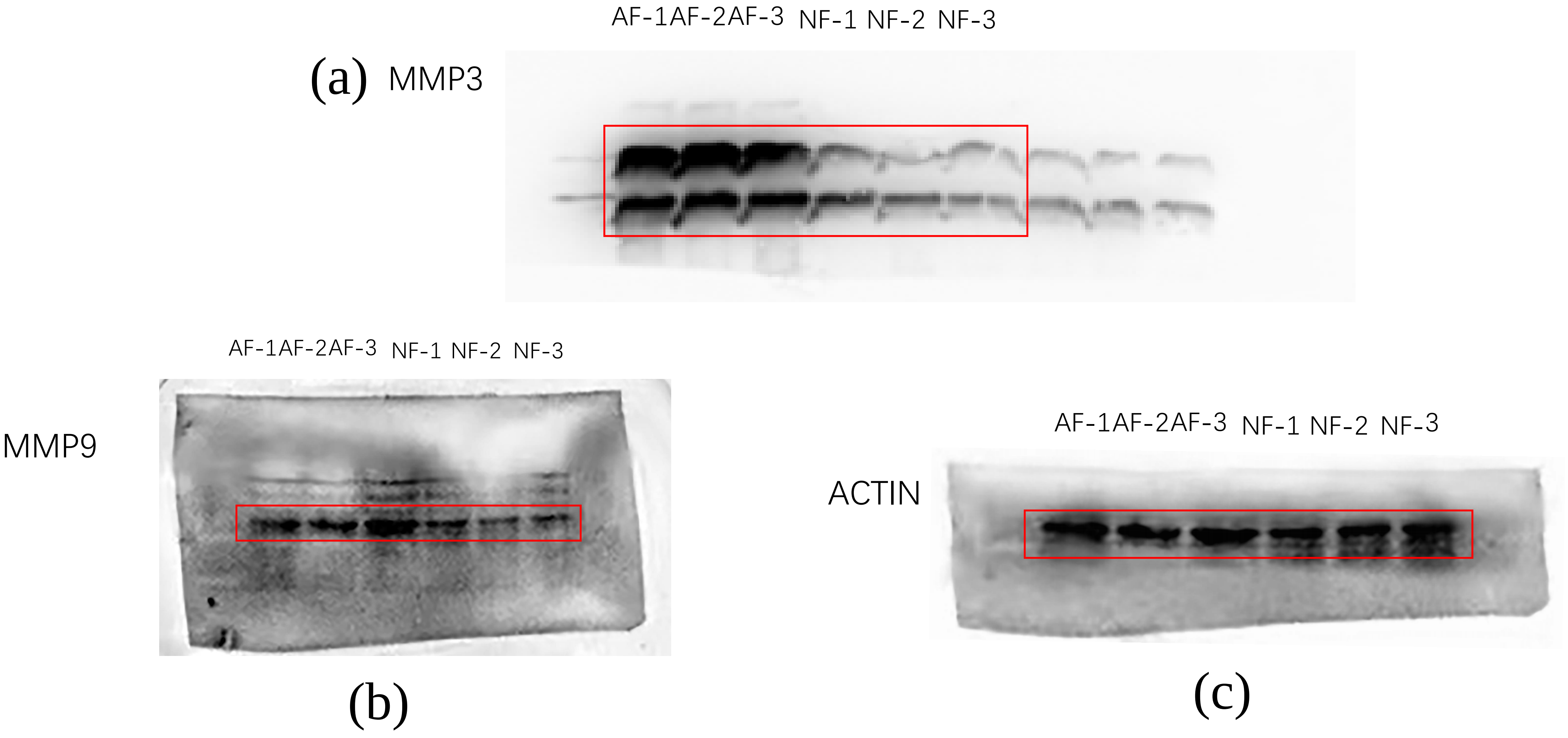

Supplement: Supplementary file 3 — Additional file 3: Fig. S3. (a) Western blotting of MMP3 protein in normal and atretic follicles. The remaining three bands, also normal follicles, were cropped out of the manuscript picture. (b) Western blotting of MMP9 protein in normal and atretic follicles. (c) Western blotting of ACTIN protein. AF-1, AF-2 and AF-3 represent the three repeats of atreated follicles, while NF-1, NF-2 and NF-3 represent the three repeats of normal follicles. The red area is the cropped area in the manuscript. The PVDF membrane was cut before incubation with antibodies. [file 12864_2022_9088_MOESM3_ESM.pdf]
